# Isolation, Identification, and Characterization of a Cellulolytic *Bacillus amyloliquefaciens* Strain SS35 from Rhinoceros Dung

**DOI:** 10.1155/2013/728134

**Published:** 2013-05-23

**Authors:** Shuchi Singh, Vijayanand S. Moholkar, Arun Goyal

**Affiliations:** ^1^Center for Energy, Indian Institute of Technology Guwahati, Guwahati, Assam 781 039, India; ^2^Department of Chemical Engineering, Indian Institute of Technology Guwahati, Guwahati, Assam 781 039, India; ^3^Department of Biotechnology, Indian Institute of Technology Guwahati, Guwahati, Assam 781 039, India

## Abstract

Cellulose hydrolyzing bacteria were isolated from rhinoceros dung and tested for clear zone formation around the colonies on the agar plates containing the medium amended with carboxymethylcellulose as a sole carbon source. Isolates were further screened on the basis of carboxymethylcellulase production in liquid medium. Out of 36 isolates, isolate no. 35 exhibited maximum enzyme activity of 0.079 U/mL and was selected for further identification by using conventional biochemical tests and phylogenetic analyses. This was a Gram-positive, spore forming bacterium with rod-shaped cells. The isolate was identified as *Bacillus amyloliquefaciens* SS35 based on nucleotide homology and phylogenetic analysis using 16S rDNA and gyrase A gene sequences.

## 1. Introduction

Cellulose, a structural carbohydrate of the plant cell wall, is an abundant and ubiquitous polymer. The use of cellulose for the second generation biofuel production involves the hydrolysis of cellulosic biomass, that is, saccharification, to form simple sugar monomers for the fermentation into bioethanol [1–3]. Cellulases are the group of enzymes involved in the conversion of cellulosic substrates to fermentable sugars. Main members of this group include endoglucanase (EC 3.2.1.4), exoglucanase or cellobiohydrolase (EC 3.2.1.91), and *β*-glucosidase (EC 3.2.1.21) [4]. The endoglucanase hydrolyzes *β*-1,4 bonds in cellulose molecule, whereas exoglucanase cleaves the ends to release cellobiose, and *β*-glucosidase converts cellobiose to glucose [5]. Several cellulase producing fungi such as *Aspergillus*, *Rhizopus*, and *Trichoderma* species [6, 7] and bacteria such as* Bacillus*,* Clostridium*,* Cellulomonas*,* Thermomonospora*, *Ruminococcus*, *Bacteroides*,* Erwinia*, and *Acetivibrio *species [8–10] have been identified. However, the isolation and characterization of novel cellulose hydrolyzing enzymes from bacteria are still a highly active research area, because bacteria have a higher growth rate than fungi, leading to greater production of enzymes [11]. Also, the habitat of bacteria covers different environmental niches, which favors the existence of versatile strains such as thermophiles [12], psychrophiles, alkaliphiles, and acidophiles. The culturable cellulase producing bacteria have been isolated from the variety of sources such as composting heaps, decaying agricultural wastes, the feces of cow [13] and elephant, gastrointestinal tract of buffalo and horse [14], soil, and extreme environments like hot-springs [15]. Cellulose degrading bacteria play an important role in energy supply for forage animals. Wahyudi et al. [14] and Varga and Kovler [16] have reported that the feed fibers were not completely converted to animal product in intensive animal farming, and 20–70% undigested cellulose was carried out with feces. Some studies have explained that the crude fiber degradation in gut is not optimal, and the fiber content of feces is still high [17], which can be utilized efficiently by microbes present in the feces of the herbivores. Rhinoceros are presumed to have an efficient system for cellulose digestion, as its main food wild grass primarily consists of cellulose. In this study, the dung of the pachyderm from Kaziranga National Park, Assam, India, has been used as the source of cellulolytic bacteria.

## 2. Materials and Methods

### 2.1. Substrate and Chemicals

Carboxymethylcellulose (CMC) (low viscosity, 50–200 cP) was procured from Sigma Aldrich (St. Louis, Mo, USA). Medium components and congo red (analytical grade) were procured from Hi-Media Pvt. Ltd., India.

### 2.2. Sample Collection

The dung sample of one-horned Indian Rhinoceros (*Rhinoceros unicornis*) was collected from its natural habitat, Kaziranga National Park, Assam, India. Presterilized spatula and plastic bags were used for sample collection, and before bacterial isolation the samples were stored at 4°C in ice box for approximately 12 h.

### 2.3. Isolation of Cellulolytic Bacteria

Dung sample (0.5 g) was suspended with 50 mL 0.85% (w/v) sterile NaCl solution in a 250 mL conical flask, which was shaken at 180 rpm for 1 h at 37°C. Serial dilutions from 10^0^ to 10^−7^ were prepared using sterilized saline solution. An aliquot of 100 *μ*L of each dilution was spread plated onto Bushnell Haas medium (BHM) [18] agar plates amended with carboxymethylcellulose (CMC) (pH 7.0) containing (g/L) CMC (10.0), K_2_HPO_4_ (1.0), KH_2_PO_4_ (1.0), MgSO_4_ *·*7H_2_O (0.2), NH_4_NO_3_ (1.0), FeCl_3_ *·*6H_2_O (0.05), CaCl_2_ (0.02), and agar (20.0) [19, 20]. The plates were incubated at 37°C for 96 h.

### 2.4. Qualitative Screening of Cellulolytic Bacteria by Plate Staining Method

Morphologically dissimilar and discrete colonies were picked from different dilution plates and streaked on separate BHM-CMC plate with grids drawn over it and incubated at 37°C for 96 h. The replica plates were also prepared separately for staining [21]. The replica plates were flooded with 0.3% congo red for 20 min. The stain was poured off, and the plates were washed with 1 M NaCl [22]. The isolates showing clear zone around the colonies were picked from master plate and further used for the enzyme production in liquid medium. The selected cultures were maintained on nutrient agar slants containing (g/L) peptone (5.0), beef extract (1.0), yeast extract (2.0), NaCl (5.0), and agar (20.0). The culture slants were stored at 4°C and subcultured every 10–15 days.

### 2.5. Quantitative Determination of Extracellular Carboxymethylcellulase (CMCase) Production

The isolates, selected on the basis of plate staining method, were grown in 50 mL enzyme production medium (at pH 7.0) containing the following components (g/L): CMC (10.0), K_2_HPO_4_ (1.0), KH_2_PO_4_ (1.0), MgSO_4_ *·*7H_2_O (0.2), NH_4_NO_3_ (1.0), FeCl_3_ *·*6H_2_O (0.05), CaCl_2_ (0.02), and yeast extract (5.0). This medium is the same as the previously used medium during isolation, with the only difference of addition of yeast extract. This addition is to provide additional nitrogen source and enhance the growth rate. 50 mL medium (containing 2% inoculum) was taken in 250 mL Erlenmeyer flask and incubated at 37°C at 180 rpm for 72 h. After every 6 h, the culture was centrifuged at 12000 g for 20 min at 4°C. The cell-free culture broth containing the crude enzyme was used for estimation of CMCase activity. Based on the higher CMCase activity (as described later), an isolate SS35 (named after its colony number) was selected for further characterization and identification. The enzyme production by the isolate SS35 was monitored with cell growth at 600 nm using UV-visible spectrophotometer (Perkin Elmer, Model lambda-45).

### 2.6. CMCase Activity Assay

The CMCase activity (U/mL) was measured by estimation of reducing sugars liberated from CMC. The enzyme assay was carried out by incubating the enzyme with CMC for 15 min at 37°C. The reaction mixture (100 *μ*L) contained 50 *μ*L of enzyme and 1.0% (w/v) final concentration of CMC in 50 mM phosphate buffer (pH 7.0). The reducing sugar was estimated by the method of Nelson and Somogyi [23, 24]. The absorbance was measured at 500 nm using a UV-visible spectrophotometer (Perkin Elmer, Model lambda-45) against a blank with d-glucose as standard. One unit (U) of cellulase activity is defined as the amount of enzyme that liberates 1 *μ*mol of reducing sugar (glucose) in 1 min at 37°C and pH 7.0.

### 2.7. Morphological and Biochemical Characterizations of the Isolate SS35

Morphological and biochemical properties of the isolate were identified, evaluated, and compared, as described in Bergey's Manual of Systematic Bacteriology [25]. The cell morphology of the selected isolate was observed under scanning electron microscope (Leo 1430 VP, Leo Electron Microscopy Ltd., Cambridge, UK) at 14 kV. Gram staining, endospore staining, and urease test were done as per standard protocol [26]. The catalase activity was determined adding few drops of 3% (v/v) H_2_O_2_ to 5 mL of 18 h grown culture [27]. The Nitrate Agar slants (M072, HiMedia) were used to test nitrate reducing property of the isolate SS35. BHM amended with starch was used for amylase activity determination. Triple Sugar Iron (TSI) slants (M021I, HiMedia) containing three sugars, namely, glucose, lactose, and sucrose, were used for acid and H_2_S production test. Acid production after carbohydrate fermentation was detected by the visible change in color from red to yellow. The temperature tolerance test was performed by growing the isolate in nutrient broth and incubating at the temperatures ranging 20°–50°C.

### 2.8. Analyses of 16S Ribosomal DNA (rDNA) and Partial Gyrase A (*gyr*A) Gene Sequences

16S rDNA and partial gyrase A gene sequencing of bacterial culture were done in Xcelris Labs Limited, Ahmedabad, India. The genomic DNA of the isolate SS35 was extracted using Qiagen DNA extraction kit and purified by QIAamp DNA Purification Kit (Qiagen) for nucleotide sequence analysis. The universal 16S rDNA primer 8F (5′ AGAGTTTGATCCTGGCTCAG 3′) and 1492R (5′ ACGGCTACCTTGTTACGACTT 3′) were used for amplification of genomic DNA by polymerase chain reaction (PCR). The *gyr*A region was amplified using the primers, p-*gyr*A-F (5′ CAGTCAGGAAATGCGTACGTCCTT 3′) and p-*gyr*A-R (5′ CAAGGTAATGCTCCAGGCATTGCT 3′) [28]. The concentration of each primer in 25 *μ*L PCR reaction mixture was 10 pmol and 1X PCR master mix (MBI Fermentas). The PCR reaction was run for 30 cycles in a Thermal Cycler (Eppendorf), and the thermal profile used for the PCR was as follows: initial denaturation at 95°C for 2 min, final denaturation at 94°C for 30 s, primer annealing at 52°C for 30 s, and extension at 72°C for 90 s. Full extension of the products was ensured by running a final cycle that included extension for 10 min at 72°C. PCR product of 5 *μ*L from each tube was mixed with 1 *μ*L of 6X gel loading dye, and this mixture was subjected to electrophoresis on 1.2% agarose gel to confirm the targeted PCR amplification. The amplified product was excised from the gel and purified using QIAamp DNA Purification Kit (Qiagen). The concentration of the purified DNA was determined, and it was subjected to automated DNA sequencing on ABI 3730xl Genetic Analyzer (Applied Biosystems, USA). The cycle sequencing was carried out using BigDye Terminator v3.1 Cycle sequencing kit following manufacturer's instructions. The cycle sequencing was carried out in a final reaction volume of 20 *μ*L using 200 *μ*L capacity PCR tube. The cycling protocol was designed for 25 cycles as follows: denaturation at 96°C for 10 s, annealing at 52°C for 5 s, and extension at 60°C for 4 min. After cycling, the extension products were purified and mixed well in 10 *μ*L of Hi-Di formamide. Eluted products were placed in a sample plate, heated at 95°C for 5 min, chilled, and loaded into autosampler of the instrument. Both the ends of the sequences were verified with the chromatogram file, and the resulted consensus sequences were used to carry out Basic Local Alignment Search Tool (BLAST) with nr database of NCBI GenBank using MEGABLAST algorithm. Multiple sequence alignment was performed by using CLUSTAL W [29], and evolutionary history was inferred using the neighbor-joining method [30]. The evolutionary distances were computed using the Kimura 2-parameters method [31], and phylogenetic analysis was carried out with MEGA4 [32].

## 3. Results and Discussion

### 3.1. Isolation and Screening of Cellulolytic Bacteria by Plate Staining Method

**Figure 1:**
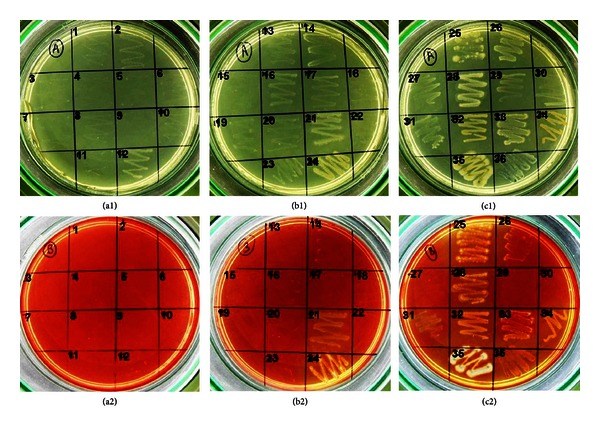
Petri plates containing 1% CMC agar incubated at 37°C for 96 h. (a1), (b1), and (c1) colonies before staining with 0.3% congo red; (a2), (b2), and (c2) colonies after staining.

**Figure 2:**
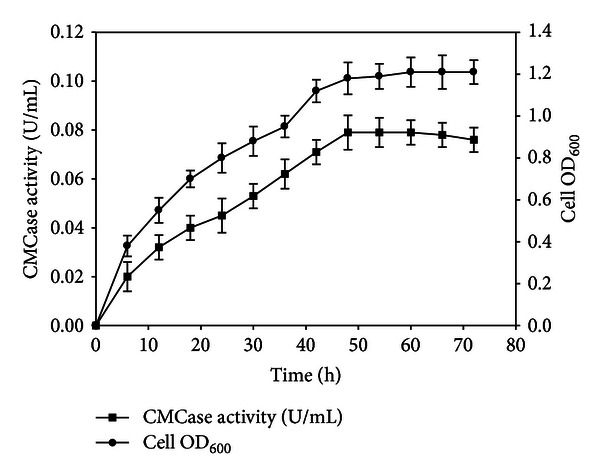
Enzyme production profile and growth curve of strain SS35.

Among 36 isolates, 9 cellulose hydrolyzing microorganisms were screened on the basis of plate staining method. The isolates (no. 21, 24, 25, 28, 31, 32, 34, 35, and 36) showed clear zone around colonies after staining the plates with congo red and destaining with 1 M NaCl as shown in Figure 1. However, plate-screening method is not quantitative because of poor correlation between enzyme activity and colony to clear zone ratio [12]. The colonies showing yellow-colored halo zones were picked from replica plate and further screened on the basis of CMCase production in liquid medium. Out of 9 isolates, isolate no. 35 exhibited maximum CMCase activity of 0.079 U/mL (details given in Table 1). This value was higher than activity of CMCase produced from some known natural isolates (expressed per mL of cell-free culture broth), for example, *Cellulomonas *sp. (0.0336 U/mL, isolated from coir retting effluents), *Micrococcus* sp. (0.0152 U/mL, isolated from coir retting effluents), *Bacillus* sp. (0.0197 U/mL, isolated from coir retting effluents), *Brevibacillus* sp. JXL (0.02 U/mL, isolated from swine waste), *Brevibacillus* sp. DUSELG12 (0.02 U/mL, isolated from gold mine), *Geobacillus* sp. DUSELR7 (0.058 U/mL, isolated from gold mine), *Geobacillus* sp. (0.0113 U/mL, isolated from sugar refinery wastewater), and *Bacillus subtilis* AS3 (0.07 U/mL, isolated from cow dung) [33–37]. Ariffin et al. [38] have reported maximum CMCase activity of 0.079 U/mL by *Bacillus pumilus* EB3 produced in a 2 L stirred tank reactor, which was equal to the CMCase activity of the isolate in this study. Thus, the isolate no. 35 was revealed to be an efficient CMCase producer species and was designated as SS35. Further analysis of this species was done as described below. The growth curve of SS35 along with CMCase production profile (Figure 2) revealed that the enzyme production was associated with cell growth and reached maximum at late log phase. Slight reduction in enzyme production after 48 h could be a consequence of instability of the enzyme at 37°C or the activity of proteases present in the crude enzyme solution.

### 3.2. Morphological and Biochemical Characterization of the Isolate SS35

**Figure 3:**
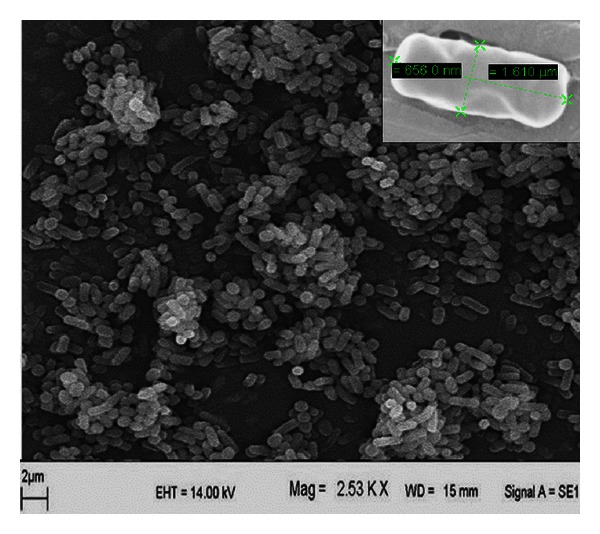
SEM micrograph for morphological characterization of isolate SS35 (inset: enlarged micrograph of a single cell).

The isolate SS35 was found to be rod-shaped cells with a width and length of 0.5–0.6 *μ*m and 1.5–1.6 *μ*m, respectively, as observed under scanning electron microscope (Figure 3). The isolate was found to be a Gram-positive, spore forming bacterium, and it gave positive test for catalase, nitrate reduction, and starch hydrolysis, whereas negative for urease and hydrogen sulfide production. The absence of black precipitate at the base of the tube indicated that hydrogen sulfide was not produced. The color of TSI agar slant was turned from red to yellow, which indicated that the bacterium was able to ferment the sugars glucose, lactose, and sucrose. The temperature tolerance test revealed that the isolate was able to grow at a wide temperature range 20°–50°C. These characteristics have been summarized in Table 2.

### 3.3. Identification on the Basis of Phylogenetic Analyses

**Figure 4:**
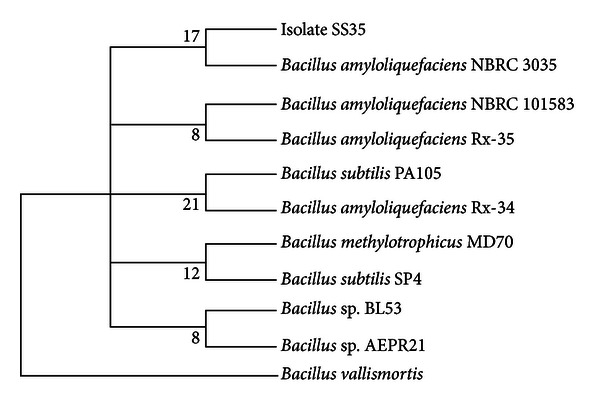
Neighbor-joining tree based on 16S rDNA sequences of *Bacillus amyloliquefaciens *SS35.

**Figure 5:**
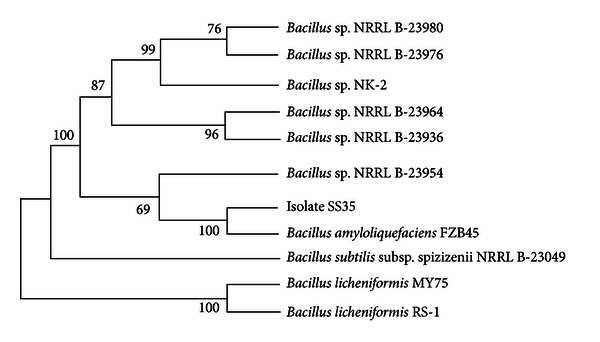
Neighbor-joining tree based on partial *gyr*A gene sequences of *Bacillus amyloliquefaciens* SS35.

The phylogenetic tree generated using 16S rDNA gene sequences of the isolate SS35 showed that the bacterium has the highest homology with *Bacillus amyloliquefaciens* NBRC 3035 (GenBank accession no.: AB679994.1) (Figure 4). The bacterial identification using 16S rDNA gene sequence is a widely practiced technique, although with limitations for the members of closely related taxa [39]. To overcome this limitation, several studies have been done, which concluded that some protein-coding genes such as RNA polymerase (*rpo*B) gene [40], RNA polymerase sigma factor (*rpo*D) gene, Gyrase B (*gyr*B) gene [41], and gyrase A (*gyr*A) gene [28] can be used for the identification of closely related taxa, because the genetic variation in protein-coding genes are much higher. Chun and Bae [28] demonstrated that the *gyr*A sequences, code for DNA gyrase subunit A, can be used for accurate identification of *Bacillus amyloliquefaciens* and related taxa including *Bacillus subtilis*, *Bacillus vallismortis*, *Bacillus mojavensis*, *Bacillus atrophaeus*, and *Bacillus licheniformis*. Therefore, in this study partial* gyr*A gene sequences have been used for the confirmation of the result obtained from 16S rDNA sequence analysis. The phylogenetic analysis using partial *gyr*A gene sequences also revealed that the isolate SS35 has the highest homology with *Bacillus amyloliquefaciens* FZB45 (GenBank accession no.: FN662840.1) [42], as shown in Figure 5. Numbers at nodes of the tree are indications of the levels of bootstrap support based on a neighbor-joining analysis of 1,500 resampled datasets. The isolate SS35 was identified as *Bacillus amyloliquefaciens* and designated as *Bacillus amyloliquefaciens* SS35. The 16S rDNA and *gyr*A gene sequences of the isolate *B. amyloliquefaciens* SS35 have been deposited in the NCBI nucleotide sequence database under the accession nos. JX674030 and KF019284, respectively.

## 4. Conclusions

Cellulose hydrolytic bacteria have been isolated from rhinoceros dung, and some potent cellulose degrading bacteria have been identified. Among all cellulolytic bacteria, isolate no. 35 exhibited maximum CMCase production and has been further characterized by biochemical methods. Bacterium has been identified as *Bacillus amyloliquefaciens *SS35 on the basis of 16S rDNA and partial gyrase A gene sequence analyses. The CMCase production has been observed to be associated with cell growth and has maxima at the late exponential phase of growth. Optimization of medium composition and fermentation parameters can further increase the cellulase production from *B. amyloliquefaciens* SS35. These attributes also make *B. amyloliquefaciens* SS35 a potential candidate in solid state fermentation for CMCase production using cellulosic biomass.

## Figures and Tables

**Table 1:** CMCase activity (U/mL cell-free culture broth) of cellulase producing isolates (after 48 h at 37°C, 180 rpm, and medium pH 7.0).

Isolate no.	CMCase activity (U/mL)
21	0.063 ± 0.008
24	0.059 ± 0.003
25	0.072 ± 0.010
28	0.073 ± 0.008
31	0.071 ± 0.006
32	0.067 ± 0.007
34	0.057 ± 0.004
35	0.079 ± 0.011
36	0.049 ± 0.003

*Values are mean ± SE (*n* = 3).

**Table 2:** Differential characteristics of *Bacillus amyloliquefaciens* SS35.

Characteristic/biochemical test	Observation
Cell shape	Rod
Cell size	0.5–0.6 *μ*m × 1.5–1.6 *μ*m,
Gram's reaction	+
Endospore formation	+
Acid production from	
Glucose	+
Lactose	+
Sucrose	+
Catalase test	+
Urease test	−
NO_3_ reduction into NO_2_	+
H_2_S production	−
Starch hydrolysis	+
Growth between 20 and 50°C	+

+: positive reaction; −: negative reaction.

## References

[1] Sun Y, Cheng J (2002). Hydrolysis of lignocellulosic materials for ethanol production: a review. *Bioresource Technology*.

[2] Martín C, López Y, Plasencia Y, Hernández E (2006). Characterisation of agricultural and agro-industrial residues as raw materials for ethanol production. *Chemical and Biochemical Engineering Quarterly*.

[3] Sanchez OJ, Cardona CA (2008). Trends in biotechnological production of fuel ethanol from different feedstocks. *Bioresource Technology*.

[4] Bayer EA, Lamed R, Himmel ME (2007). The potential of cellulases and cellulosomes for cellulosic waste management. *Current Opinion in Biotechnology*.

[5] Bhat MK, Bhat S (1997). Cellulose degrading enzymes and their potential industrial application. *Biotechnology Advances*.

[6] Murashima K, Nishimura T, Nakamura Y (2002). Purification and characterization of new endo-1,4-*β*-D-glucanases from Rhizopus oryzae. *Enzyme and Microbial Technology*.

[7] Saito K, Kawamura Y, Oda Y (2003). Role of the pectinolytic enzyme in the lactic acid fermentation of potato pulp by Rhizopus oryzae. *Journal of Industrial Microbiology and Biotechnology*.

[8] Roboson LM, Chambliss GH (1989). Celluases of bacterial origin. *Enzyme and Microbial Technology*.

[9] Lee YJ, Kim BK, Lee BH (2008). Purification and characterization of cellulase produced by *Bacillus amyoliquefaciens* DL-3 utilizing rice hull. *Bioresource Technology*.

[10] Kim BK, Lee BH, Lee YJ, Jin IH, Chung CH, Lee JW (2009). Purification and characterization of carboxymethylcellulase isolated from a marine bacterium, *Bacillus subtilis* subsp. *subtilis* A-53. *Enzyme and Microbial Technology*.

[11] Lynd LR, Weimer PJ, Van Zyl WH, Pretorius IS (2002). Microbial cellulose utilization: Fundamentals and biotechnology. *Microbiology and Molecular Biology Reviews*.

[12] Maki M, Leung KT, Qin W (2009). The prospects of cellulase-producing bacteria for the bioconversion of lignocellulosic biomass. *International Journal of Biological Sciences*.

[13] Akhtar N, Sharma A, Deka D, Jawed M, Goyal D, Goyal A (2012). Characterization of cellulase producing *Bacillus* sp. for effective degradation of leaf litter biomass. *Environmental Progress and Sustainable Energy*.

[14] Wahyudi A, Cahyanto MN, Soejono M, Bachruddin Z (2010). Potency of lignocellulose degrading bacteria isolated from Buffalo and horse gastrointestinal tract and elephant dung for feed fiber degradation. *Journal of the Indonesian Tropical Animal and Agriculture*.

[15] Doi RH (2008). Cellulases of mesophilic microorganisms: cellulosome and noncellulosome producers. *Annals of the New York Academy of Sciences*.

[16] Varga GA, Kovler ES (1997). Microbial and animal limitation to fiber digestion and utilization. *Journal of Nutrition*.

[17] Krause DO, Denman SE, Mackie RI (2003). Opportunities to improve fiber degradation in the rumen: microbiology, ecology, and genomics. *FEMS Microbiology Reviews*.

[18] Bushnell DL, Haas HF (1941). The utilization of certain hydrocarbons by microorganisms. *Kansas Agricultural Experiment Station*.

[19] Lo YC, Saratale GD, Chen WM, Bai MD, Chang JS (2009). Isolation of cellulose-hydrolytic bacteria and applications of the cellulolytic enzymes for cellulosic biohydrogen production. *Enzyme and Microbial Technology*.

[20] Atlas RM (2004). *Handbook of Microbiological Media*.

[21] Ruijssenaars HJ, Hartmans S (2001). Plate screening methods for the detection of polysaccharase-producing microorganisms. *Applied Microbiology and Biotechnology*.

[22] Teather RM, Wood PJ (1982). Use of Congo red-polysaccharide interactions in enumeration and characterization of cellulolytic bacteria from the bovine rumen. *Applied and Environmental Microbiology*.

[23] Nelson N (1944). A photometric adaptation of the Somogyi method for the determination of glucose. *Journal of Biological Chemistry*.

[24] Somogyi M (1945). A new reagent for the determination of sugars. *Journal of Biological Chemistry*.

[25] Boone DR, Garrity GM, Castenholz RW, Brenner DJ, Krieg NR, Staley JT (2001). Genus *Bacillus*. *Bergey’s Manual of Systematic Bacteriology: The Firmicutes*.

[26] Cappuccino JC, Sherman N (2004). *Microbiology—A Laboratory Manual*.

[27] Kannan N (2002). *Laboratory Manual in General Microbiology*.

[28] Chun J, Bae KS (2000). Phylogenetic analysis of *Bacillus subtilis* and related taxa based on partial gyrA gene sequences. *Antonie van Leeuwenhoek*.

[29] Thompson JD, Higgins DG, Gibson TJ (1994). CLUSTAL W: improving the sensitivity of progressive multiple sequence alignment through sequence weighting, position-specific gap penalties and weight matrix choice. *Nucleic Acids Research*.

[30] Saitou N, Nei M (1987). The neighbor-joining method: a new method for reconstructing phylogenetic trees. *Molecular biology and evolution*.

[31] Kimura M (1980). A simple method for estimating evolutionary rates of base substitutions through comparative studies of nucleotide sequences. *Journal of Molecular Evolution*.

[32] Tamura K, Dudley J, Nei M, Kumar S (2007). MEGA4: Molecular Evolutionary Genetics Analysis (MEGA) software version 4.0. *Molecular Biology and Evolution*.

[33] Immanuel G, Dhanusha R, Prema P, Palavesam A (2006). Effect of different growth parameters on endoglucanase enzyme activity by bacteria isolated from coir retting effluents of estuarine environment. *International Journal of Environmental Science and Technology*.

[34] Liang Y, Yesuf J, Schmitt S, Bender K, Bozzola J (2009). Study of cellulases from a newly isolated thermophilic and cellulolytic *Brevibacillus* sp. strain JXL. *Journal of Industrial Microbiology and Biotechnology*.

[35] Rastogi G, Muppidi GL, Gurram RN (2009). Isolation and characterization of cellulose-degrading bacteria from the deep subsurface of the Homestake gold mine, Lead, South Dakota, USA. *Journal of Industrial Microbiology and Biotechnology*.

[36] Tai SK, Lin HPP, Kuo J, Liu JK (2004). Isolation and characterization of a cellulolytic *Geobacillus thermoleovorans* T4 strain from sugar refinery wastewater. *Extremophiles*.

[37] Deka D, Bhargavi P, Sharma A, Goyal D, Jawed M, Goyal A (2011). Enhancement of cellulase activity from a new strain of *Bacillus subtilis* by medium optimisation and analysis with various cellulosic substrates. *Enzyme Research*.

[38] Ariffin H, Abdullah N, Kalsom MSU, Shirai Y, Hassan MA (2006). Production and characterization of cellulase by *Bacillus pumilus* EB3. *International Journal of Engineering and Technology*.

[39] Fox GE, Wisotzkey JD, Jurtshuk P (1992). How close is close: 16S rRNA sequence identity may not be sufficient to guarantee species identity. *International Journal of Systematic Bacteriology*.

[40] Kim BJ, Lee SH, Lyu MA (1999). Identification of mycobacterial species by comparative sequence analysis of the RNA polymerase gene (rpoB). *Journal of Clinical Microbiology*.

[41] Yamamoto S, Harayama S (1998). Phylogenetic relationships of Pseudomonas putida strains deduced from the nucleotide sequences of gyrB, rpoD and 16S rRNA genes. *International Journal of Systematic Bacteriology*.

[42] Idriss EE, Makarewicz O, Farouk A (2002). Extracellular phytase activity of *Bacillus amyloliquefaciens* FZB45 contributes to its plant-growth-promoting effect. *Microbiology*.

